# Telomerase Variant A279T Induces Telomere Dysfunction and Inhibits Non-Canonical Telomerase Activity in Esophageal Carcinomas

**DOI:** 10.1371/journal.pone.0101010

**Published:** 2014-07-01

**Authors:** Yuwei Zhang, Rodrigo Calado, Mahadev Rao, Julie A. Hong, Alan K. Meeker, Bogdan Dumitriu, Scott Atay, Peter J. McCormick, Susan H. Garfield, Danny Wangsa, Hesed M. Padilla-Nash, Sandra Burkett, Mary Zhang, Tricia F. Kunst, Nathan R. Peterson, Sichuan Xi, Suzanne Inchauste, Nasser K. Altorki, Alan G. Casson, David G. Beer, Curtis C. Harris, Thomas Ried, Neal S. Young, David S. Schrump

**Affiliations:** 1 Thoracic Surgery Section, Thoracic and GI Oncology Branch; National Cancer Institute, Bethesda, Maryland, United States of America; 2 National Heart, Lung, and Blood Institute, Bethesda, Maryland, United States of America; 3 Departments of Pathology and Oncology, Johns Hopkins University of Medicine, Baltimore, Maryland, United States of America; 4 Laboratory of Cellular Oncology, National Cancer Institute, Bethesda, Maryland, United States of America; 5 Laboratory of Experimental Carcinogenesis, National Cancer Institute, Bethesda, Maryland, United States of America; 6 Section of Cancer Genomics, National Cancer Institute, Bethesda, Maryland, United States of America; 7 Comparative Molecular Cytogenetics Core Facility, National Cancer Institute, Frederick, Maryland, United States of America; 8 Department of Thoracic Surgery, Weill Cornell Medical Center, New York, New York, United States of America; 9 Department of Surgery, University of Saskatchewan, Saskatoon, Saskatchewan, Canada; 10 Section of Thoracic Surgery, University of Michigan Medical Center, Ann Arbor, Michigan, United States of America; 11 Laboratory of Human Carcinogenesis, National Cancer Institute, Bethesda, Maryland, United States of America; University of Science and Technology of China, China

## Abstract

**Background:**

Although implicated in the pathogenesis of several chronic inflammatory disorders and hematologic malignancies, telomerase mutations have not been thoroughly characterized in human cancers. The present study was performed to examine the frequency and potential clinical relevance of telomerase mutations in esophageal carcinomas.

**Methods:**

Sequencing techniques were used to evaluate mutational status of *telomerase reverse transcriptase (TERT)* and *telomerase RNA component (TERC)* in neoplastic and adjacent normal mucosa from 143 esophageal cancer (EsC) patients. MTS, flow cytometry, time lapse microscopy, and murine xenograft techniques were used to assess proliferation, apoptosis, chemotaxis, and tumorigenicity of EsC cells expressing either wtTERT or TERT variants. Immunoprecipitation, immunoblot, immunofluorescence, promoter-reporter and qRT-PCR techniques were used to evaluate interactions of TERT and several TERT variants with BRG-1 and β-catenin, and to assess expression of cytoskeletal proteins, and cell signaling. Fluorescence in-situ hybridization and spectral karyotyping techniques were used to examine telomere length and chromosomal stability.

**Results:**

Sequencing analysis revealed one deletion involving *TERC (TERC del 341-360)*, and two non-synonymous *TERT* variants [A279T (2 homozygous, 9 heterozygous); A1062T (4 heterozygous)]. The minor allele frequency of the A279T variant was five-fold higher in EsC patients compared to healthy blood donors (p<0.01). Relative to wtTERT, A279T decreased telomere length, destabilized TERT-BRG-1-β-catenin complex, markedly depleted β-catenin, and down-regulated canonical Wnt signaling in cancer cells; these phenomena coincided with decreased proliferation, depletion of additional cytoskeletal proteins, impaired chemotaxis, increased chemosensitivity, and significantly decreased tumorigenicity of EsC cells. A279T expression significantly increased chromosomal aberrations in mouse embryonic fibroblasts (MEFs) following Zeocin™ exposure, as well as Li Fraumeni fibroblasts in the absence of pharmacologically-induced DNA damage.

**Conclusions:**

A279T induces telomere dysfunction and inhibits non-canonical telomerase activity in esophageal cancer cells. These findings warrant further analysis of A279T expression in esophageal cancers and premalignant esophageal lesions.

## Introduction

Telomeres are highly evolved nucleoprotein structures, which function to maintain and protect chromosomal ends [1]. Telomeric DNA contains long tandem hexameric repeats (TTAGGG), capped by shelterin proteins (TRF1, TRF2, RAP1, TPP1, POT1, TIN2), which prevent activation of DNA double strand break repair at chromosomal ends [2], [3]. With each cell replication, telomere length decreases until a critical point is reached (Hayflick limit), whereby further telomere attrition induces replicative senescence or apoptosis [4]. Via repeat addition processivity mechanisms, human telomerase ribonucleoprotein complex successively adds hexameric repeats to chromosomal ends [5], [6], thereby slowing telomere attrition; this complex is composed of two copies of telomerase reverse transcriptase (TERT), and two copies of its RNA template (TERC), as well as additional proteins such as N0P10, NHP2, GAR and dyskerin, which bind to TERC to stabilize the complex [2].

Increasing evidence indicates that telomere dysfunction contributes to the pathogenesis of a variety of human cancers by mechanisms, which have not been fully elucidated [2], [7]–[10]. Recently a patient with a history of Barrett's esophagus presented to the National Cancer Institute for treatment of a locally advanced esophageal adenocarcinoma. Additional evaluation revealed pancytopenia, the etiology of which could not be ascertained despite extensive evaluation, and liver cirrhosis without portal hypertension. The family history was notable for anemia, biliary cirrhosis, and esophageal cancer. The patient underwent esophagectomy with final pathology revealing T3N0M0 (Stage IIB) adenocarcinoma. Post-operatively, the patient developed progressive hepatic insufficiency, and died approximately four months later. Subsequent analysis revealed a germ-line deletion in telomerase RNA component (TERC del 341–360) [11]; this loss-of-function mutation was also identified in the proband's son, who at 30 years of age exhibited premature aging, mild anemia, and early cirrhosis. The present study was undertaken to examine the frequency and potential clinical relevance of telomerase complex mutations in sporadic esophageal cancers.

## Materials and Methods

### Ethics Statement

All human tissues were procured on IRB-approved protocols. All mouse experiments were approved by the National Cancer Institute Animal Care and Use Committee, and were in accordance with the NIH Guide for Care and Use of Laboratory Animals.

### Patient samples

Genomic DNA was isolated as described [12] from snap-frozen esophageal cancers and adjacent normal mucosa from 80 patients undergoing potentially curative resections at the National Cancer Institute, University of Michigan, and Dalhousie University. In addition, genomic DNA was extracted from formalin-fixed paraffin embedded (FFPE) tissues from 63 esophageal cancer patients from Cornell University Medical Center, using PicoPure DNA Extraction Kit (Qiagen; Valencia, CA), and later purified with DNeasy Blood & Tissue Kit (Qiagen). PCR products from snap-frozen tissues were purified with a QIAquick PCR purification kit (Qiagen), followed by direct sequencing as described [13]. PCR products from FFPE samples were analyzed by pyrosequencing techniques using primers listed in Table S1.

### Cell lines and reagents

Esophageal adenocarcinoma lines, NCI-SB-EsC1 (EsC1) and NCI-SB-EsC2 (EsC2) were established from two patients with Stage IV esophageal adenocarcinoma. These cell lines exhibit HLA and cytokeratin expression profiles identical to the respective primary tumors, and have been continuously passaged for >4 years. The TERT/TERC deficient VA-13 lung fibroblast line [11] was provided by Dr. Neal Young (NIH). HCT116, HeLa, and mouse embryonic fibroblast (MEF) cell lines were obtained from American Type Culture Collection (Manassas, VA). All cells were maintained in RPMI 1640 media at 37°C in 5% CO_2_. Li Fraumeni fibroblasts (MDAH087) were generously provided by Michael Tainsky (Karmanos Cancer Institute, Detroit, MI), and were cultured as described [14]. The proteasome inhibitors, MG132 and ALLN were obtained from Sigma (Allentown, PA), reconstituted in DMSO, and stored at −20°C. Cisplatin and paclitaxel were purchased from the Clinical Center Pharmacy at the NCI.

### Cell Proliferation Assays

EsC1 cells (4×10^3^ cells per well) and EsC2 cells (8×10^3^ cells per well) were plated in 96-well plates in 100 µL media. Cell viability was quantitated by MTS colorimetric techniques using the Cell Titer 96 Aqueous One Solution Cell Proliferation Assay (Promega; Madison, WI). For chemosensitivity experiments, responses to cisplatin or paclitaxel were plotted as fractions of viable cells relative to untreated controls. Each experiment was performed in triplicate at least twice.

### Annexin V-FITC assay

Apoptosis was assessed using the Annexin V-FITC kit (Abcam; Cambridge, MA) according to vendor protocols.

### Telomerase activity by telomerase repeats amplification protocol

Two micrograms of pcDNA3-Flag-TERC or -Terc 341–360 del were co-transfected with either 2 µg of pcDNA3-Flag vector, pcDNA3-Flag- wtTERT, or -A279T into VA-13 cells at 60 percent confluency in 6-well polystyrene dishes using Superfect Transfection Reagent (Qiagen), according to manufacturer's instructions. Telomerase activity in transfected cells was determined using the fluorescent telomerase repeat amplification kit (TRAPeze XL; Chemicon; Temecula, CA) as previously described [11].

### Telomere length assay

Mean telomere length in esophageal cancer cells constitutively expressing wtTERT, A279T, or vector control sequences were analyzed by quantitative polymerase chain reaction (qPCR) techniques. PCR was conducted in triplicate in a Rotor-Gene Q real-time instrument with the Rotor-Gene SYBR Green Kit (Qiagen). The telomere length for each sample was determined using the telomere to single copy gene ratio (T/S ratio) with the calculation of the DCt[Ct(telomere)/Ct(single gene)]. The T/S ratio for each sample was normalized to the mean T/S ratio of reference sample [2_(DCtx_DCtr) 1/4 2_DDCt], which was used for the standard curve, both as a reference sample and as a validation sample [15].

### Generation of TERT and mutant stable cells

pLenti4/TO/V5-hTERT and pLentiviral4/TO/V5-A279T were generated using reagents and protocols provided by Invitrogen (Carlsbad, CA), and primers listed in Table S1. The only difference between wtTERT and A279T sequences is a single nucleotide (G to A) change, resulting in the substitution of threonine for alanine at codon 279. Empty pcDNA 3.0 vector as well as pcDNA3 vectors expressing wtTERT, A279T TERT, G260D TERT, A1062T TERT, or TERC del 341–360 were provided by Neal Young. These vectors were used to transduce/transfect EsC1, EsC2, HeLa or HCT116 cells followed by selection with Zeocin for lentiviral transduced cells, or G418 for cells transfected with pcDNA vectors. Constitutive expression of TERT or A279T was assayed by real time PCR using primers listed in Table S1. Genotyping of transfected/transduced cells was confirmed by sequencing and PyroMark techniques. Unless otherwise mentioned, stable transductants/transfectants were used for all experiments. Target gene expression was confirmed by qRT-PCR and immunoblot techniques.

### PCR Superarray and quantitative reverse transcription-PCR (qRT-PCR)

Effects of wtTERT and A279T expression on Wnt, tumor suppressor and stem cell gene expression were analyzed using human Q-PCR arrays (SA Bioscience; Frederick, MD). Confirmatory quantitative RT-PCR experiments were performed using primers listed in Table S1.

### Immunoblot, Immunoprecipitation, and Immunofluorescence

Total cellular proteins were extracted using the RIPA buffer lysis kit (Millipore; Billerica, MA) supplemented with 1X protease inhibitor (Roche, Inc., Indianapolis, IN), and 1 mmol/L phenylmethylsulfonyl fluoride. Cell lysates were resolved on 4–20% Tris glycine gels (Invitrogen), transferred to PVDF membranes, and incubated overnight with primary antibodies listed in Table S2. HeLa cells were transiently transfected with wtTERT, A279T and G260D mutants and immunoprecipitated with anti-TERT (Rocklands), BRG-1 (Millipore) and β-catenin (Abcam; Cambridge, MA). Immunoblot signals were detected using appropriate horseradish peroxidase–conjugated secondary antibodies, and SuperSignal West Pico Chemiluminescent Substrate (Pierce Biotechnology; Rockford, IL).

For immunofluorescence experiments, 1×10^5^ cells were grown on LAB-TEK II slides and fixed for 5 min with ice-cold ethanol. Slides were blocked with 1% BSA in PBS for 30 min. Cells were incubated for one hour in blocking solution with primary antibodies listed in Table S2, washed and then incubated for 30 min with appropriate secondary antibodies. Immunofluorescence analysis of F-actin and vinculin was performed using the Actin Cytoskeleton/Focal Adhesion Staining Kit (Millipore) and secondary antibody (Goat anti-Mouse IgG, (H+L) FITC Conjugated;Millipore) according to vendor protocols. Slides were mounted in VECTASHIELD Mounting Medium with DAPI. A Zeiss LSM 710 confocal microscope (25x) was used to evaluate all slides except for vinculin images, which were recorded using a Nikon A1 Confocal Microscope with the objective of Plan Apo 20X VC 0.75NA. Images were acquired under the same conditions and displayed at the same scale for comparison.

### Luciferase Promoter-Reporter Transient Transfection Experiments

1×10^5^ HeLa cells were plated per well in 24-well plates. After 24 hours, cells were transiently co-transfected with empty vector, wtTERT and A279T with the T-cell factor (TCF) responsive vector TOPFlash and the TCF mutant vector FOPFlash (Millipore) using Lipofectamine 2000 (Invitrogen). Approximately 24 hours later, cells were lysed and assayed for luciferase activity using the dual luciferase reporter assay (Promega) according to vendor instructions. Renilla luciferase activity was used as a control to normalize inter-sample variability.

### Chemotaxis and Time-lapse Video Microscopy

Chemotaxis of EsC1and ExC2 cells was performed as described [16] with minor modifications. Briefly, EsC1 and EsC2 cells (10^6^/ml) were plated in serum free RPMI-1640 media on collagen type IV-coated microslides (Ibidi; Prospect, IL), and left to adhere for 4 hours at room temperature. Microslide reservoirs were then filled with serum free media, and 18 µl of chemoattractant (10% FBS) was added; 15 minutes later, cancer cell migration was monitored using a Zeiss LSM 510 or 710 NLO confocal microscope. AIM or ZEN Imaging software (Zeiss) was used for time-lapse imaging. Phase-contrast images were captured every 15 minutes. Image J Plugin was used to manually track cells, and characterize chemotaxis from the captured images. Average total movement of all cells within the experimental time was defined as the center of mass. Rayleigh test for inhomogeneity of cell distribution was determined with Ibidi Chemotaxis Tool software.

### Murine Xenograft Experiments

EsC2- wtTERT and EsC2-A279T cells were trypsinized, washed in HBSS, suspended in sterile PBS at a concentration of 1×10^6^ cells per 100 µL, and inoculated in contralateral flanks of athymic nude mice. Tumor size and take were recorded biweekly. Tumors were excised, weighed, and processed for additional studies.

### Telomere-Specific FISH Analysis

FFPE tissue sections (5 µm thickness) from paired tumors were placed on the same slide to ensure that FISH conditions were identical for paired samples. Deparaffinized slides were hydrated, steamed for 20 minutes in citrate buffer, dehydrated and hybridized with a Cy3-labeled peptide nucleic acid (PNA) probe complementary to the mammalian telomere repeat sequence ([N-terminus to C-terminus]). As a positive control for hybridization efficiency, a FITC-labeled PNA probe having specificity for human centromeric DNA repeats (CENP-B binding sequence) was also included in the hybridization solution. Confocal images were sequentially acquired with Zeiss ZEN 2009 software on a Zeiss LSM 710 Confocal System (Carl Zeiss Inc, Thornwood, NY) with a Zeiss Observer Z1 inverted microscope and Chameleon IR laser tuned to 760 nm, a 25 mW Argon visible laser tuned to 488 nm and a 15 mW DPSS laser tuned to 561 nm. A 63x Plan-Apochromat 1.4 NA oil immersion objective was used, and digital images were 512×512 pixels with 0.264 µm pixel size. Emission signals after sequential excitation of DAPI, GFP, and Rhodamine by the 760 nm, 488 nm or 561 nm laser lines were collected with a BP 419–485 nm filter, BP 495–534 nm filter, and BP 568–624 nm filter, respectively, using individual photomultipliers. Images were acquired under the same conditions and displayed at the same scale for comparison.

### Spectral Karyotype (SKY) Analysis

SKY probes were prepared as described [17], [18]. Parental murine MEF-1 cells and human Li Fraumeni cells or respective cells stably transfected with control vectors, wtTERT, or A279T were grown in normal media (DMEM for MEF-1 cells and MEM for Li Fraumeni cells), and metaphases were arrested by overnight incubation with Colcemid prior to harvest. MEF-1 cells were also treated with Zeocin™ (100 µg/ml) for three days as described [19] to induce double strand breaks. Thereafter, debris was removed, and viable cells were washed with HBSS, and incubated in normal media overnight. The following day, metaphases were arrested, and SKY analysis of mouse and human chromosomes was performed. The images of MEF-1 cells were acquired with a spectral cube system (Applied Spectral Imaging, Migdal Haemek, Israel) attached to a fluorescence microscope (DMRXA, Leica, Wetzlar, Germany), and the emission spectrum was measured with a custom – made triple-band-pass filter (Chroma Technology, Bellows Falls, VT). Spectral images of the hybridized metaphases with Li Fraumeni cells were acquired using a SD300 SpectraCubeTM system (Applied Spectral Imaging Inc., CA) mounted on top of an epifluorescence microscope Axioplan 2 (Zeiss). Approximately 10–15 metaphase spreads per sample were analyzed, and scored for numerical and structural aberrations. Human cells were analyzed following the nomenclature rules presented in ISCN (2009). For mouse cells, chromosome analysis followed established nomenclature rules: http://www.informatics.jax.org/mgihome/nomen/gene.shtml.

### Statistical analysis

Differences in the frequencies of coding-sequence variations between samples from patients and those from controls were evaluated by means of Fisher's exact test, considering a p value <0.05 as statistically significant. T test was used to analyze results from all other experiments except chemotaxis assays described above.

## Results

### Frequency of TERC and TERT Mutations in Esophageal Cancers

Except for the one observed in the proband, no TERC mutations were identified among 54 patients. Direct sequencing analysis revealed two non-synonymous TERT variants (A279T and A1062T) among these 54 patients; one homozygous and 4 heterozygous A279T variants were detected, whereas one heterozygous A1062T variant was identified. To confirm and extend these observations, pyrosequencing techniques were used to analyze the frequency of A279T and A1062T in 89 additional esophageal carcinoma specimens. The previous homozygous A279T variant was confirmed with this approach (Figure S1). Several additional A279T and A1062T variants were detected in these specimens. In all cases in which a TERT variant was identified in esophageal cancer, the same variant was detected in matched normal esophageal mucosa.

The overall frequencies of A279T and A1062T variants identified in 143 esophageal cancers are summarized in Table 1. The minor allele frequency (mAF) of A279T [SNP database rs61748181] in esophageal cancers (∼5%) was significantly higher than that previously observed in a large number of healthy adult blood donors (0.9%), or individuals with aplastic anemia [20]; [21], and was comparable to that previously reported for patients with bone marrow failure and dyskeratosis congenita (DC) [22]. In contrast, the mAF of A1062T in esophageal cancers was not significantly different than that previously observed in healthy blood donors [21].

**Table 1:** Mutations of TERT in Esophageal Cancers among 143 Cases.

		Esophageal CA		Healthy controls (21)	Aplastic anemia (21)	Bone marrow failure (22)
		Adenocarcinoma	Squamous cell carcinoma			Dyskeratosis congenita
Total		117	26	528	200	80
A279T†	Homozygous	2	0	0	0	1
	Heterozygous	6	3	10	6	5
	mAF*	4.27%	5.77%	0.90%	1.50%	4.37%
	p value**			<0.01	<0.05	NS***
A1062T‡	Homozygous	0	0	0		
	Heterozygous	3	1	7		
	mAF*	1.28%	1.92%	0.66%		
	p value**			NS***		

† codon 279 GCC/ACC (Ala/Thr).

‡ codon 1062 GCC/ACC (Ala/Thr)].

*mAF (minor allele frequency): frequency of the less frequent allele in a given population.

**p value: compared to both esophageal cancers combined. Fisher's exact test.

***NS: not significant.

### Effects of A279T on Proliferation of Esophageal Cancer Cells

**Figure 1:**
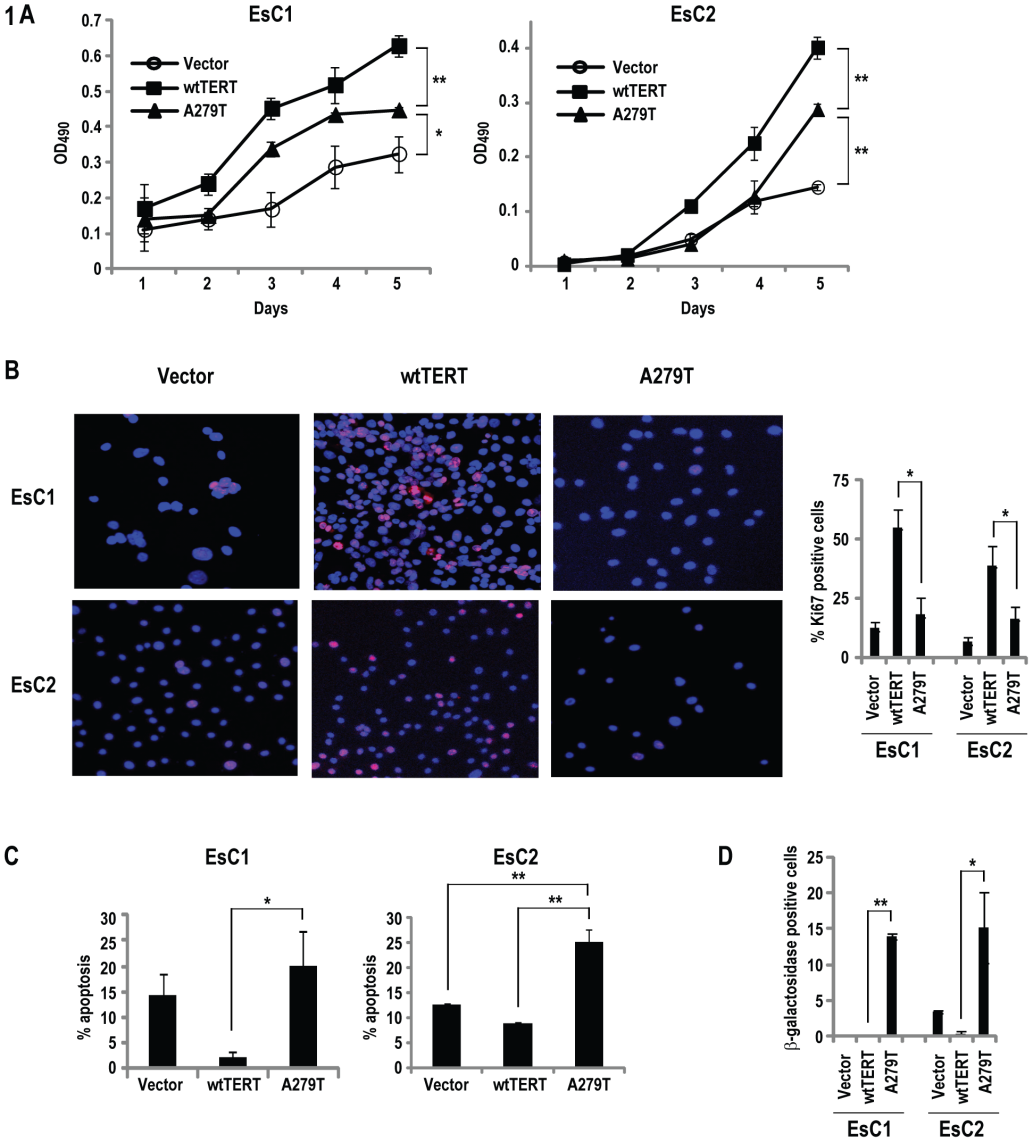
A279T inhibits proliferation of esophageal cancer cells. (*p<0.05; **p<0.01). A. MTS assay demonstrating inhibition of EsC1 (left) and EsC2 (right) proliferation by A279T relative to wtTERT. B. Immunofluorescence analysis (left panel) with corresponding summary (right panel) of Ki67 expression in esophageal cancer cells expressing wtTERT or A279T (Red: Ki67; blue: DAPI). EsC1 and EsC2 cells expressing A279T exhibit decreased Ki67 levels relative to respective cells expressing wtTERT. C. Annexin V-FITC assay demonstrating A279T-induces apoptosis in EsC2 but not EsC1 cells. Results are expressing as mean ± SD of triplicate experiments. D. Graphic summarization of immunofluorescence analysis of β-galactosidase expression in EsC1 and EsC2 following constitutive expression of wtTERT or A279T. Red: β-galactosidase; blue: DAPI.

The fact that the mAF of A279T in esophageal cancers was approximately five-fold higher than that observed in peripheral blood from healthy donors suggested that this variant might contribute to the pathogenesis of these malignancies. As such, a series of experiments were performed to examine if A279T expression modulated the malignant phenotype of esophageal cancer cells. EsC1 and EsC2 cells, which exhibit low level wtTERT and TERC expression (Table S3), were stably transduced with lentiviral vectors encoding A279T or wtTERT, or control sequences. MTS assays revealed that EsC1 and EsC2 cells expressing A279T (EsC1-A279T and EsC2-A279T, respectively) grew significantly slower than cells constitutively expressing wtTERT (EsC1-TERT, and EsC2-TERT, respectively), yet faster than vector controls (Figure 1A). Immunofluorescence experiments demonstrated that Ki67 levels in EsC1-TERT and EsC2-TERT cells were significantly higher than those observed in respective vector controls, consistent with increased proliferation mediated by TERT over-expression. In contrast Ki67 levels in EsC1-A279T and ESC2-A279T were modestly but insignificantly higher than those in vector controls, and significantly lower than those observed in respective TERT-over-expressers. Annexin V experiments demonstrated a significant increase in apoptotic index in EsC1-A279T and EsC2-A279T cells relative to respective cells over-expressing wtTERT (Figure 1C). Subsequent immunohistochemistry experiments demonstrated that β-galactosidase levels were significantly higher in A279T-transduced esophageal cancer cells relative to respective TERT-transduced or vector control cells (Figure 1D). These preliminary findings suggested that the A279T amino acid substitution simultaneously induced apoptosis and senescence, which attenuated the proliferative effects of telomerase over-expression in esophageal cancer cells.

### Effects of A279T on Telomerase Activity and Telomere Length

**Figure 2:**
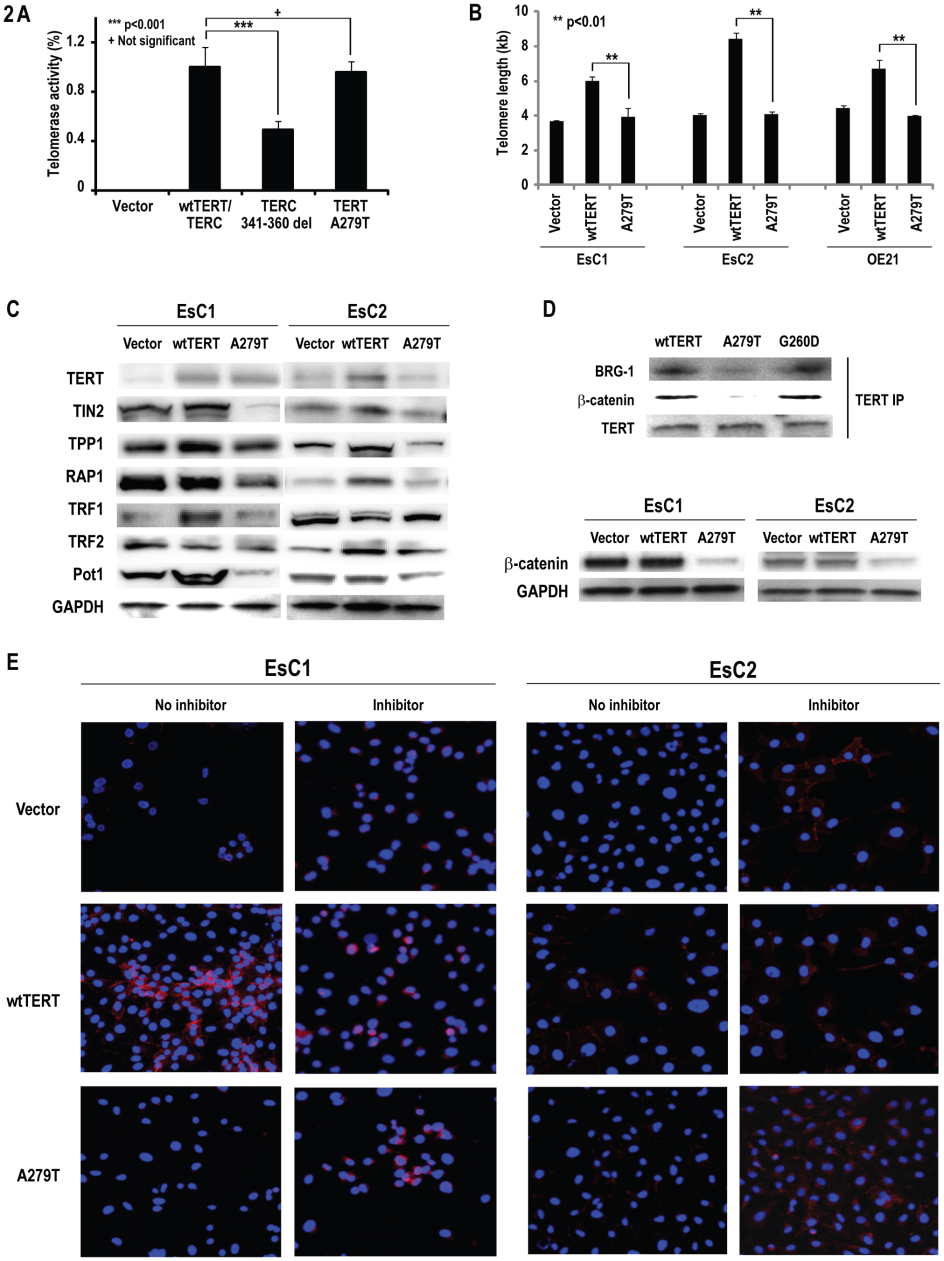
A279T down-regulates β-catenin independent of telomerase activity. A. Telomerase enzymatic activity of TERT and TERC mutations in VA13 cells, measured by TRAPeze assay. Telomerase activity is defined as 100% of wtTERT. B. Quantitative PCR analysis of telomere lengths in EsC1, EsC2 and OE21 esophageal cancer cells transfected with empty vector, wtTERT, or A279T-TERT. C. Immunoblot analysis of TERT and related shelterin protein levels in EsC1 and EsC2 cells transduced with wtTERT, A279T-TERT or empty vector. Expression of A279T depletes several shelterin proteins in esophageal cancer cells. D. Upper panel: Immunoprecipitation experiments demonstrating that A279T disrupts TERT-BRG-1-β-catenin complex. This phenomenon was not seen in cells expressing G260D. Lower panel: immunoblot experiments demonstrating decreased β-catenin levels in EsC1 and EsC2 expressing A279T. E. Representative immunofluorescence analysis of β-catenin expression in EsC1 and EsC2 cells cultured in normal media in the presence or absence of proteasome inhibitors (red: β-catenin; blue: DAPI).

Additional experiments were performed to examine if A279T expression modulated telomerase catalytic activity and telomere length in esophageal cancer cells. In initial experiments, vectors containing A279T or wtTERT were co-transfected with either TERC del 341–360 (TERCdel) or wtTERC into TERT/TERC-deficient VA-13 cells; telomerase catalytic activity was measured in cell lysates. Results of this analysis are depicted in Figure 2A. Consistent with previous observations [11], TERCdel significantly reduced telomerase enzymatic activity relative to wild-type TERC. In contrast, A279T did not appear to significantly diminish telomerase catalytic activity under these experimental conditions.

In subsequent experiments, quantitative PCR techniques were used to examine mean telomere lengths in EsC1 and EsC2 cells stably transduced (>1 year) with wtTERT, A279T, or control vectors. Parental EsC1 and EsC2 exhibit moderate levels of TERC and relatively low endogenous levels of TERT (Table S3). Results of this analysis are depicted in Figure 2B. Mean telomere lengths in A279T-transduced EsC cells were significantly shorter than those observed in wtTERT-transduced cells, and were, in fact, similar to those observed in respective vector controls. Immunoblot analysis using an antibody that recognized wtTERT as well as A279T, demonstrated that the differences in mean telomere lengths observed in A279T-relative to wtTERT transduced esophageal cancer cells were not attributable to consistent differences in telomerase protein levels (Figure 2C). Additional immunoblot experiments (Figure 2C) demonstrated that relative to EsC1-TERT and EsC2-TERT, or respective vector controls, EsC1-A279T and EsC2-A279T cells exhibited decreased levels of several shelterin proteins including POT1, which binds to single stranded telomeric 3′ overhangs, as well as TIN2, which together with TPP1, connects POT1 to TRP1 to regulate telomere length, and prevent telomeres from activating non-homologous end joining (NHEJ) or other DNA double strand break repair pathways [3]. Collectively, these results suggest that A279T-TERT disrupts primary as well as secondary/tertiary telomere structure in esophageal cancer cells.

### Effects of A279T on Non-canonical TERT Activities

Recent studies have demonstrated that in addition to TERC dependent (canonical) elongation of telomeres, TERT enhances cell proliferation and immortalization by non-canonical mechanisms including direct interactions with BRG-1 and β-catenin [23]. As such, additional studies were undertaken to ascertain if the TERT A279T variant affected non-canonical TERT activity in esophageal cancer cells. Briefly, HeLa cells were transiently transfected with control vectors, wtTERT, or A279T; immunoprecipitation techniques were then utilized to examine interactions of TERT with BRG-1 and β-catenin. HeLa cells were chosen for these experiments because relative to EsC1 or EsC2, these cells exhibit high transfection efficiency, as well as abundant levels of endogenous BRG-1 [24]. Results of these experiments are shown in Figure 2D, upper panel. Compared to wtTERT-transfected HeLa (TERT-HeLa) cells, immunoprecipitates from A279T-HeLa cells had much lower levels of BRG-1 and β-catenin following pull-down with an anti-TERT antibody. Similarly, TERT and BRG-1 levels were lower in β-catenin immunoprecipitates from A279T-HeLa relative to TERT-HeLa cells. Lastly, β-catenin and TERT levels were lower in BRG-1 immunoprecipitates from A279T-HeLa cells relative to TERT-HeLa cells. These results were not observed in HeLa cells transfected with G260D, a TERT variant frequently detected in hematologic malignancies [25], [26], which is in the same region of TERT where A279T occurs (Figure 2D). Furthermore, these results were not observed in HeLa cells transfected with A1062T, another TERT variant associated with hematologic disorders [25] (Figure S2). Consistent with these findings, immunoblot experiments demonstrated markedly decreased β-catenin levels in EsC1-A279T as well as EsC2-A279T cells relative to respective vector controls, or EsC cells constitutively expressing wtTERT (Figure 2D; lower panel). Quantitative RT-PCR experiments demonstrated that changes in β-catenin levels mediated by A279T in these cells did not coincide with consistent alterations in β-catenin mRNA levels (data not shown).

Since free intracellular β-catenin levels are tightly regulated by the cytoplasmic APC/Axin destruction complex [27], additional experiments were performed to examine the effects of proteosome inhibitors in esophageal cancer cells constitutively expressing A279T or wtTERT. Immunofluorescence analysis demonstrated that MG132 and ALLN attenuated A279T-mediated decreases in β-catenin levels in EsC1 and EsC2 cells (Figure 2E). Collectively, these findings suggest that A279T destabilizes the BRG-1-TERT-β-catenin complex, resulting in depletion of β-catenin via proteosomal degradation in esophageal cancer cells.

### Effects of A279T on Canonical Wnt Signaling

**Figure 3:**
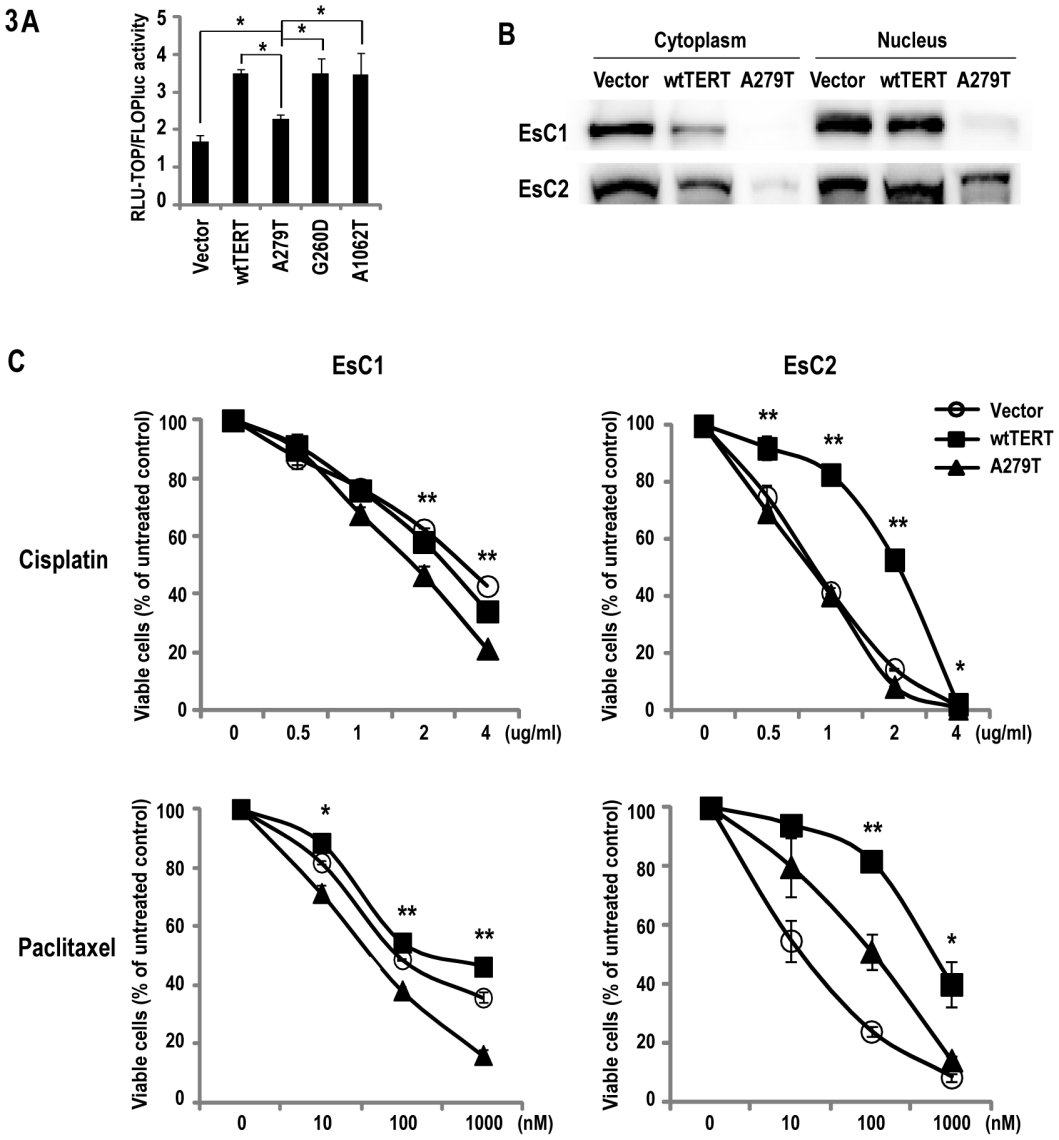
Effects of A279T on Wnt signaling and chemosensitivity in cancer cells. *p<0.05, **p<0.01. A. TOP-flash promoter-reporter assay demonstrating that relative to wtTERT, A279T inhibits Wnt signaling in HeLa cells. This phenomenon was not seen in cells expressing G260D or A1062T. B. Immunoblot of β-catenin levels in cytoplasmic and nuclear extracts of EsC1 and EsC2 cells following constitutive expression of wtTERT or A279T-TERT. C. MTS assay demonstrating that relative to EsC1 and EsC2 cells expressing wtTERT, EsC1 and EsC2 cells expressing A279T are more sensitive to cisplatin (2 day treatment) and paclitaxel (3 hour treatment) measured on day 3.

β-catenin is a critical mediator of canonical Wnt signaling [28], translocating from the plasma membrane to the nucleus to activate target genes [29]. Therefore, additional experiments were performed to examine if A279T modulated Wnt activity in cancer cells. Briefly, HeLa cells transiently expressing either control vector, wtTERT, A279T, G260D or A1062T TERT sequences were transfected with either TOP-FLASH or FOP-FLASH promoter reporters. Once again, HeLa cells were chosen for these experiments due to high transduction efficiency. Results of these experiments are summarized in Figure 3A. As expected, TCF luciferase activity was significantly increased in cells transfected with wtTERT relative to control vector. Comparable increases in luciferase activities were observed in HeLa cells expressing G260D as well as A1062T telomerase variants. In contrast, whereas A279T-HeLa cells also exhibited higher TCF promoter activity compared to vector controls, luciferase levels in A279T-HeLa cells were significantly lower than those observed in wtTERT, G260D or A1062T transfectants.

To extend these observations, immunoblot and qRT-PCR array experiments were performed to examine the effects of A279T expression on Wnt signaling and associated pathways in esophageal cells. Immunoblot experiments demonstrated depletion of β-catenin in nuclear as well as cytoplasmic extracts from EsC1-A279T and to a lesser extent EsC2-A279T cells relative to respective TERT-transduced cells, or vector controls (Figure 3B). Focused qRT-PCR arrays and confirmatory qRT-PCR experiments (Table 2) demonstrated that relative to wtTERT, A279T mediated repression of several Wnt-related genes in EsC1 and/or EsC2 cells including cyclin D1, a well-established target of canonical Wnt signaling [30]. Furthermore, consistent with recent observations that β-catenin directly regulates TERT expression [31], [32], endogenous TERT mRNA levels were lower in A279T-EsC1 and A279T-EsC2 cells relative to EsC1 and EsC2 cells over-expressing wtTERT. Additional experiments revealed that a variety of mediators of DNA damage response and apoptosis/senescence including BRCA1, BRCA2, p57, caspase 8, TNF, FAS, IL-6 and IL-8 were induced, whereas JunB was repressed in EsC-1 and/or EsC2 cells expressing A279T relative to wtTERT.

**Table 2:** Gene expression levels of A279T-transduced cells normalized to wtTERT-transduced cells.

	EsC1-A279T			EsC2-A279T		
	Mean	SEM	p value	Mean	SEM	p value
**TERT**	0.1	0.01	5.0E-05	0.4	0.05	5.5E-03
**CCND1**	0.1	0.01	7.5E-06	ND*		
**JUNB**	0.1	0.01	1.5E-03	0.7	0.08	3.3E-02
**Dkk-1**	381.8	175.96	9.8E-02	2.8	0.38	1.9E-02
**CASP8**	1.1	0.26	8.5E-01	3.5	0.42	6.4E-03
**p57**	2.2	0.19	1.0E-02	1.7	0.15	1.9E-02
**IL-6**	9.6	1.00	1.0E-03	25.7	1.53	8.6E-05
**IL-8**	1.3	0.06	5.4E-03	5.6	0.22	3.6E-05
**BRCA1**	4.4	0.13	5.0E-05	1.8	0.24	4.6E-02
**BRCA2**	4.9	0.33	5.1E-04	2.4	0.32	1.5E-02
**JUNB**	0.1	0.01	1.5E-03	0.7	0.08	3.3E-02
**TNF**	0.3	0.16	5.6E-02	28.3	2.10	2.0E-04

*ND: Not detected.

### Effects of A279T on Chemosensitivity of Esophageal Cancer Cells

Because telomerase activity, telomere length, and Wnt/β-catenin signaling appear to modulate chemoresistance in cancer cells [33]–[36], additional experiments were performed to ascertain if A279T affected sensitivity of esophageal cancer cells to cisplatin and paclitaxel, two agents typically used to treat esophageal carcinomas in clinical settings. Preliminary experiments were undertaken to optimize drug exposure conditions and timing of viability assays. As shown in Figure 3C, cisplatin as well as paclitaxel mediated dose-dependent cytotoxicity in EsC1 as well as EsC2 cells. Relative to cells expressing wtTERT, EsC1-A279T and EsC2-A279T appeared more sensitive to cisplatin and paclitaxel. This phenomenon was more impressive in EsC2 cells; A279T abolished TERT-mediated resistance to cisplatin, and significantly diminished TERT-mediated resistance to paclitaxel.

### Effects of A279T on Cytoskeletal Integrity and Chemotaxis in Cancer Cells

**Figure 4:**
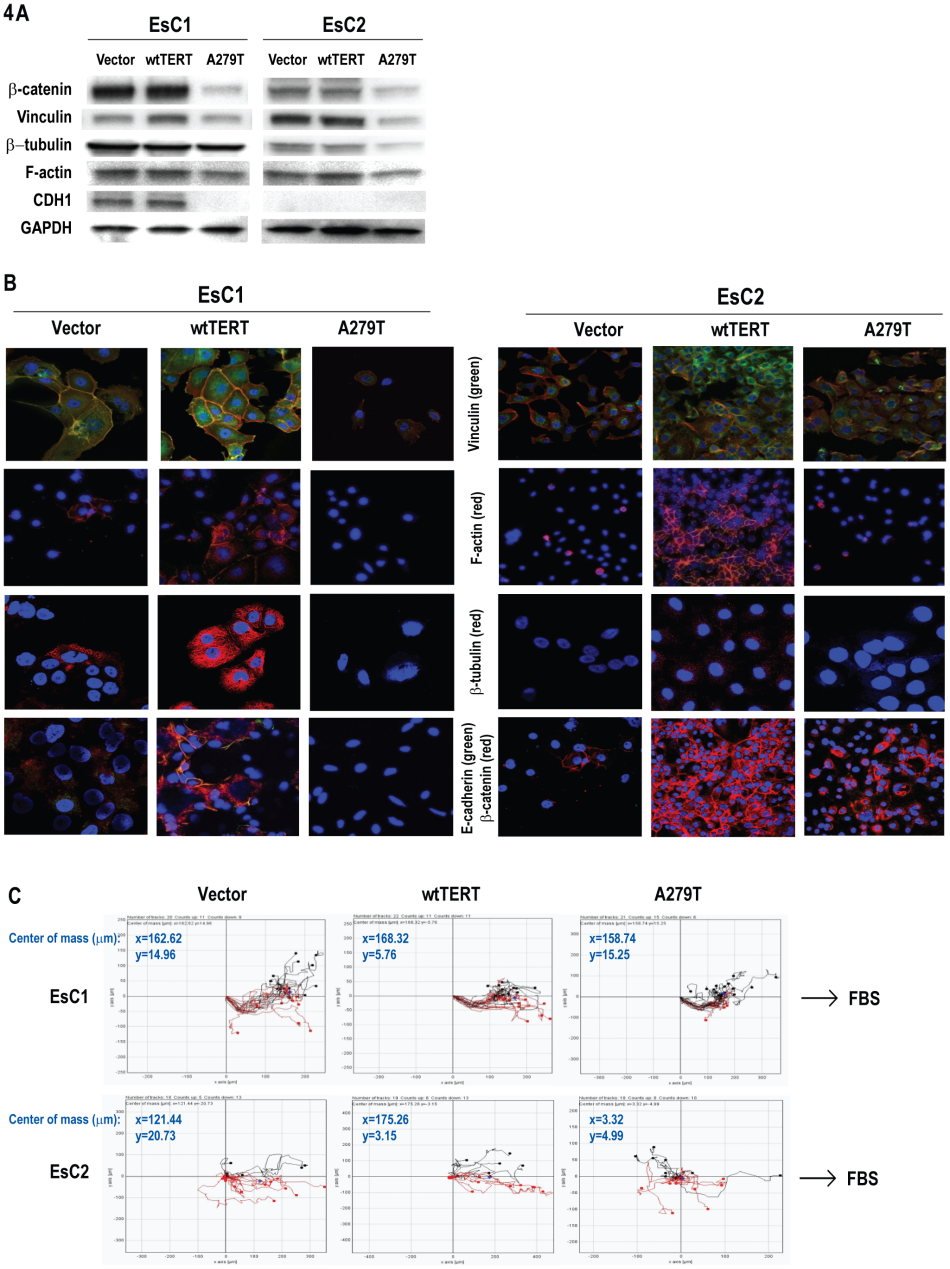
A279T depletes cytoskeletal proteins and impairs chemotaxis in esophageal cancer cells. A. Immunoblot analysis depicting down-regulation of β-catenin, vinculin, β-tubulin, F-actin and CDH1 in EsC1 and EsC2 cells expressing A279T-TERT relative to wtTERT or control vectors. B. Immunofluorescence analysis demonstrating that A279T disrupts cytoskeletal integrity in EsC1 and EsC2 cells as evidenced by decreased vinculin, F-actin, β-tubulin, E-cadherin and β-catenin expression. (Blue: DAPI). C. Results of time-lapse video microscopy demonstrating that EsC1 and EsC2 cells expressing A279T exhibit impaired chemotaxis relative to parental cells, or cells expressing wtTERT.

β-catenin, α-catenin and p120 interact with the intracellular domain of E-cadherin at the plasma membrane, thereby stabilizing adherens junctions, and connecting the cadherin-catenin complex to microtubules, as well as actin and actin-associated proteins such as F-actin, vinculin, and formin-1 [37]. As such, additional experiments were performed to ascertain if expression of A279T affected cytoskeletal organization in cancer cells. Although some variability was noted between lines, immunoblot experiments (Figure 4A) revealed that relative to cells constitutively expressing wtTERT or control vectors, EsC1- and EsC2- A279T cells not only had decreased β-catenin levels, but also exhibited reduced expression of vinculin, β-tubulin, F-actin, and CDH1. Immunofluorescence experiments (Figure 4B) confirmed results of immunoblot analyses.

Because A279T appeared to disrupt cytoskeletal organization, additional studies were undertaken to directly examine if A279T affected cell motility. Briefly, EsC1 and EsC2 cells constitutively expressing control vector, wtTERT, or A279T were placed in chamber slides and time lapse microscopy techniques [16] were used to evaluate chemotaxis in response to mitogen. Representative results are depicted in Figure 4C. EsC1-TERT and EsC2-TERT, as well as respective vector controls exhibited chemotaxis in response to FBS. In contrast, chemotaxis was significantly impaired in EsC1-A279T cells, and was completely abolished in EsC2-A279T cells (p<0.05 for A279T vs. wtTERT).

### Effects of A279T on Tumorigenicity of Esophageal Cancer Cells

**Figure 5:**
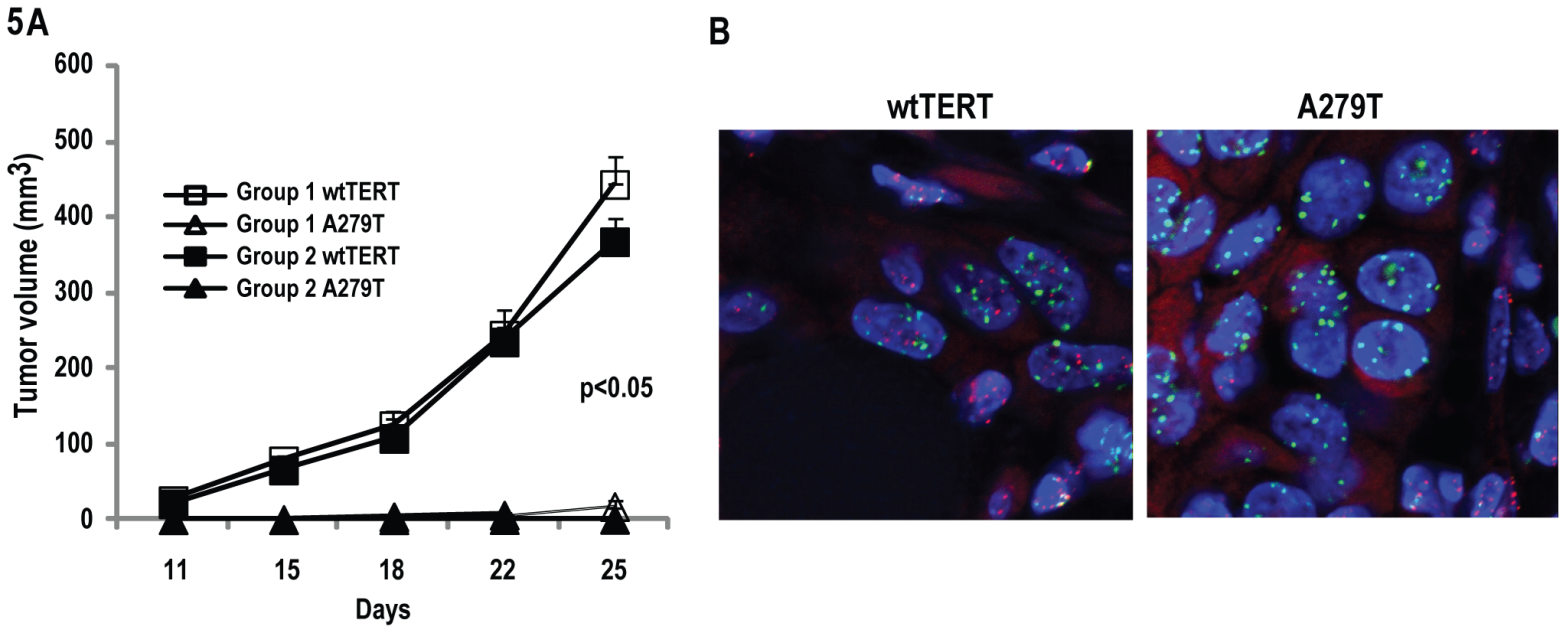
Effects of A279T on tumorigenicity and telomere length of esophageal cancer cells in vivo. A. A279T significantly inhibits growth of subcutaneous EsC2 xenografts in athymic nude mice. B. Representative results of telomere-specific FISH analysis of ExC2 xenografts depicting shortened telomere length in xenografts from EsC2-A279T cells compared to those derived from EsC2-TERT cells; Red: telomere; green: centromere; blue: DAPI.

Additional experiments were performed to ascertain if expression of A279T affected tumorigenicity of esophageal cells. Briefly, EsC2 cells constitutively expressing wtTERT or A279T were inoculated subcutaneously into athymic nude mice. Representative results of two independent experiments are depicted in Figure 5A. EsC2-A279T cells exhibited only 60% tumor take compared to 100% for EsC2-TERT cells. Furthermore, volumes and masses of EsC2-A279T xenografts were significantly less than EsC2-TERT tumors (p<0.05). Similar experiments using EsC1 cells were not possible since parental EsC1 cells are not tumorigenic in nude mice (data not shown).

Fluorescence in-situ hybridization (FISH) experiments were performed to examine if the effects of A279T on tumorigenicity coincided with decreased telomere length in esophageal cancer cells. Representative results of these telomere FISH experiments are depicted in Figure 5B. Murine chromosomes in stromal cells exhibited relatively weak centromeric signals (green) due to the fact that the probe set used for FISH had greater affinity for human centromeric repeats. On the other hand, chromosomes in mouse stromal cells exhibited intense red staining due to very long telomeres [38]. EsC2-TERT cells exhibited strong green centromeric signals as well as bright red telomeric staining. Whereas EsC2-A279T xenografts also exhibited strong centromeric signals, these cells lacked red telomeric staining, indicative of short telomeres.

### Effects of A279T on Chromosomal Integrity in Normal Cells

**Figure 6:**
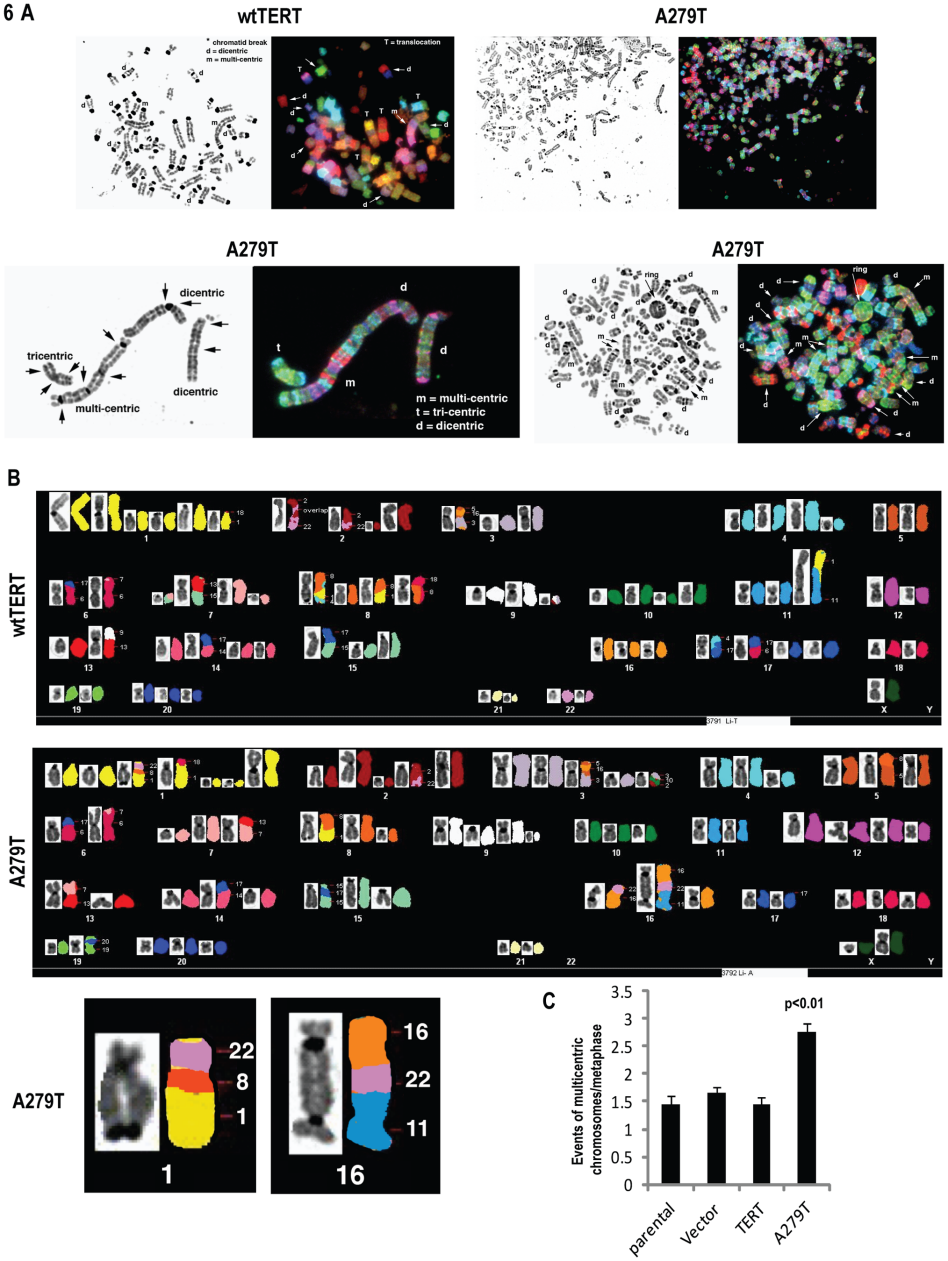
Effects of A279T on genomic stability in normal cells. A. SKY assay demonstrating that A279T-induces genomic instability in Zeocin™-treated MEF-1 cells. Upper panel: translocations, dicentric, and rearranged chromosomes are present in cells expressing A279T compared to wtTERT. Lower left panel: a multi-centric chromosome observed in cells harboring A279T. Lower right panel: a ring chromosome is formed and every chromosome is rearranged in cells transfected with A279T. See text for additional details. B. Upper panel: representative results of SKY analysis Li Fraumeni fibroblasts constitutively expressing wtTERT or A279T-TERT. Lower panel: close-up of chromosomes 1 and 16. C. Summary of results of two independent experiments demonstrating that A279T expression increases genomic instability in Li Fraumeni cells.

Results of experiments described above strongly suggested that A279T expression inhibits the malignant phenotype of esophageal cancer cells. On the other hand, the fact that the MAF of A279T was significantly higher in esophageal cancer patients relative to healthy blood donors suggested that expression of this telomerase variant predisposes to malignancy. In order to reconcile these discrepant observations, experiments were undertaken to examine if A279T affected chromosome integrity in normal cells. In initial experiments, mouse embryonic fibroblasts (MEF) stably transfected with control vectors, wtTERT, or A279T were cultured for 72 h in normal media with or without Zeocin to induce double strand breaks. Cells were then evaluated by spectral karyotyping (SKY) techniques. Representative results of these experiments are depicted in Figure 6A. Structural aberrations included translocations (t), deletions (del), dicentric (d), and multi-centric (m; three or more centromeres) chromosomes, rings, and other chromosome-breakage exchanges. In all untreated cells, ring chromosomes and evidence of chromosome-breakage were rarely detected. Untreated parental, vector-control and wtTERT-transfected MEFs consisted predominantly of hypo-tetraploid cells (<4 n) exhibiting few clonal numerical and structural aberrations. There were more translocation events and more hyper-tetraploid cells (>4 n) in A279T-MEF cells, but the difference between A279T and control MEF cells was not statistically significant, possibly due to the low number of cells analyzed (average of 15 cells per sample), as well as the fact that murine chromosomes have very long telomeres [38]. In contrast, A279T-MEFs treated with Zeocin exhibited significantly higher numbers of structural aberrations relative to Zeocin treated parental, vector control, or wtTERT transfected cells (p = 0.000249, p = 0.001, p = 0.0105, respectively); Zeocin-treated A279T- MEFs had approximately twice the number of rings and multi-centric chromosomes, with virtually every chromosome involved in translocations.

To further examine this issue, additional SKY experiments were performed using Li Fraumeni fibroblasts stably transfected with control vectors, wtTERT, or A279T. Results of two independent experiments, which were performed without Zeocin, are depicted in Figure 6B. The numbers of chromosomal abnormalities in Li Fraumeni fibroblasts constitutively expressing wtTERT were not significantly different than those observed in parental cells or vector controls. In contrast, Li Fraumeni fibroblasts constitutively expressing A279T exhibited approximately two-fold higher numbers of multicentric chromosomes, with numerous translocations (p<0.01), indicative of genomic instability.

## Discussion

Mutations or sequence variants within telomerase complex genes have been linked to a variety of benign inflammatory conditions such as pulmonary fibrosis [2] and biliary cirrhosis [11], inherited bone marrow failure syndromes [2], as well as aging [39] and cancer [2], [40]. Telomere dysfunction evidenced by loss of telomere length has been identified in myelodysplasia [15] as well as premalignant lesions in breast, pancreas, prostate, lung, colon and esophagus [7]. In malignancy, telomere attrition induces telomere recombination [8] and chromosomal rearrangements through breakage/fusion/bridge mechanisms [9], as well as tetraploidization [10], resulting in activation of DNA damage response and early crisis. Inactivation of Rb and p53 tumor suppressor pathways enables preneoplastic cells with telomere dysfunction to emerge from crisis [4]; subsequent activation of TERT by a variety of mechanisms prevents further telomere shortening during late stages of malignant transformation, and in established cancers [31], [32], [41], [42]. Approximately 10–15% of human cancers lack detectable telomerase activity; in these neoplasms telomere length is maintained by telomerase independent, alternative lengthening of telomeres (ALT) mechanisms [43]. Although frequently observed in sarcomas and CNS malignancies, ALT appears to be quite uncommon in epithelial malignancies [44].

In the present study we sought to examine the frequency and potential clinical relevance of telomerase complex mutations in sporadic esophageal carcinomas after identifying a unique germline TERC deletion in a patient with Barrett's adenocarcinoma [11]. Although we observed no additional TERC mutations, our analysis identified a telomerase variant (A279T) that occurred nearly five-fold more frequently in esophageal cancer patients compared to healthy blood donors; the frequency of A279T variant expression in esophageal cancers exceeds that of recently described ALK mutations in non-small cell lung cancers [45]. The fact that A279T was observed in tumor as well as corresponding normal esophageal mucosa strongly suggests that this was a germline variant; however, because we did not have corresponding peripheral blood samples to analyze, our results cannot exclude the possibility that A279T was a mutation acquired during field cancerization [46]. Additional experiments revealed that A279T decreased telomere length and destabilized the BRG1-TERT-β-catenin complex, depleting β-catenin in esophageal cancer cells. Relative to wtTERT, A279T mediated growth inhibition and apoptosis/senescence in-vitro, disrupted cytoskeletal integrity, markedly impaired chemotaxis, increased chemosensitivity and significantly reduced tumorigenicity of esophageal cancer cells. To the best of our knowledge, these experiments are the first to identify a telomerase variant in a human malignancy that simultaneously disrupts canonical as well as non-canonical telomerase activities.

Whereas perpetual replicative capacity is directly linked to canonical telomerase activities [47], other aspects of cancer cell biology appear attributable to telomerase independent functions of TERT, including transcriptional modulation of Wnt β-catenin signaling [23], or RNA polymerase activity when TERT is complexed with the RNA component of mitochondrial RNA processing endoribonuclease (RMRP) [48]. Indeed, recent observations that constitutive expression of β-catenin increases cell cycle progression, and promotes full malignant transformation in TERT-immortalized human fetal hepatocytes, with up-regulation of genes mediating invasion and angiogenesis [49], attest to the significance of non-canonical telomerase activities during initiation and progression of cancer. In our study we observed that esophageal cancer cells expressing A279T had short telomeres relative to cells constitutively expressing wtTERT; these findings are consistent with observations by Vulliamy et al [22] that leukocytes from individuals with A279T genotype have short telomeres. However, our current results have not precisely defined the mechanisms by which A279T induces telomere dysfunction in esophageal carcinomas. A279T occurs in a region of TERT that is not essential for in-vitro activity of telomerase [22], which may explain our inability to observe effects of A279T on telomerase catalytic activity using TRAPeze assays. In this regard our findings are consistent with previously published studies demonstrating no significant decrease in telomerase catalytic activity by TRAPeze or direct primer extension assays [50], [51]. Conceivably, deficient repeat addition processivity [5], [6] could contribute to inhibition of telomere length in esophageal cancer cells expressing A279T; however, recent studies by Zaug et al [52] using well-established rabbit reticulocyte lysate experiments have demonstrated no effect of this TERT variant on processivity functions of telomerase. Alternatively, A279T may destabilize interactions of TERT with other telomerase complex proteins, and as suggested by our immunoblot experiments, impair chromosomal capping by shelterin proteins [26], [53]. Studies are in progress to further characterize the effects of A279T on telomere biology in normal and cancer cells.

The fact that TERT not only interacts with and stabilizes β-catenin [23], [54], but also is a direct target of β-catenin signaling [31], [32], indicates that highly complex and interdependent regulatory networks mediate canonical and non-canonical telomerase activities in cancer cells. As such, precisely ascribing various phenotypic alterations in esophageal cancer cells to effects of A279T on canonical versus non-canonical telomerase activities may be quite difficult, particularly in light of recent observations that TERT over-expression increases growth of primary epithelial cells via processes, which are independent of TERT catalytic activity, chromosomal capping, or Wnt β-catenin signaling [55]. For instance, we observed that β-catenin was markedly depleted in cancer cells expressing A279T, and that A279T attenuated TERT-mediated chemoresistance in esophageal cancer cells. Whereas inhibition of Wnt/β-catenin signaling has been shown to sensitize oropharyngeal and prostate cancer cells to cisplatin and paclitaxel, respectively [36], [56], more recent studies [33], [34] suggest that telomere length determines chemosensitivity in cancer cells. Collectively, these findings, together with recent observations that telomerase regulates heterochromatin structure within centromeres and transposons via interactions with BRG-1 and nucleostemin [57], suggest that the effects of A279T expression in cancer cells are pleiotropic and highly complex, and in all likelihood contingent on genetic/epigenetic landscapes. Current efforts are focused on identification of cancer cell lines endogenously expressing A279T to further examine these issues.

Recent elegant experiments have demonstrated that telomere dysfunction disrupts alternative splicing of multiple genes- some of which encode cytoskeletal proteins, and induces senescence in normal fibroblasts [58]. These observations together with our findings that A279T induced senescence, disrupted cytoskeletal integrity and markedly impaired chemotaxis in esophageal cancer cells raise the possibility that esophageal cancers expressing A279T might have low metastatic potential, hence more favorable clinical behavior. Unfortunately, the relatively small sample size and incomplete data regarding stage, response to therapy and survival of the patients whose tissues were used for this study precluded any assessment of the prognostic or predictive significance of A279T expression in esophageal cancers. Such analysis using a larger sample size and tissues linked to complete clinical databases should be undertaken if possible to confirm our initial observations, and define the clinical relevance of A279T expression in esophageal carcinomas.

Telomere shortening correlates with genomic instability [59] and progression to adenocarcinoma [60] in Barrett's esophagus- a chronic condition in which the squamous epithelia in the distal esophagus is replaced by proliferating, intestinal-type columnar epithelial cells in the context of gastro-esophageal reflux [61]. These findings suggest that telomere dysfunction occurs early during esophageal carcinogenesis. In our study we observed that relative to wtTERT, A279T significantly increased chromosomal aberrations in MEFs with wt p53 following exposure to Zeocin; A279T induced chromosomal instability in p53 deficient Li Fraumeni-fibroblasts in the absence of DNA damage; similar chromosomal aberrations have been identified during oncogene-mediated immortalization of human esophageal epithelial cells [62]. As such, our findings provide a potential mechanism (genomic instability) by which A279T could facilitate esophageal carcinogenesis- particularly in the context of p53 mutations, which are frequently observed in esophageal cancers and their precursor lesions [63]. Consistent with this notion, we have recently detected p53 mutations in 3 of 4 esophageal cancer samples expressing A279T. Insufficient genomic DNA prevented us from fully evaluating p53 status relative to A279T expression in the remaining tissue samples. These issues are the focus of ongoing studies in our laboratory.

Several recent studies suggest that leukocyte telomere length is an indicator of total body aging, and that decreased leukocyte telomere length coincides with predisposition to cancer [64], [65]. Of particular relevance regarding our current study are observations by Risques and colleagues [66] that patients with Barrett's esophagus with leukocyte telomeres that are short relative to their age have significantly increased risk of esophageal adenocarcinomas. These findings suggest that germline mutations involving telomerase complex predispose to esophageal cancers. Unfortunately, we were unable to access samples from the Risques study to determine if germline A279T expression correlates with esophageal cancer risk. Such studies should be undertaken if possible to ascertain if A279T is a potential biomarker of progression to cancer in patients with Barrett's esophagus.

It is counter-intuitive that A279T-mediated perturbations of telomerase appear to be oncogenic in non-transformed cells, yet tumor suppressive in esophageal cancer cells. However, these paradoxical observations are consistent with recent studies demonstrating that constitutive telomerase dysfunction inhibits metastatic progression in murine breast and prostate cancer models [67], [68].

Because this was not a case control study, it is possible that our analysis over-estimated the apparent enrichment of A279T in esophageal cancer patients. Indeed, depending on which database is queried, the mAF of A279T ranges from 0.9% in a large pool of healthy adult blood donors (528 individuals) including Caucasians, Blacks, Latinos and Asians [21], to 2.2% in patients with diverse pathologic conditions including idiopathic pulmonary fibrosis, aplastic anemia, acute myeloid leukemia, and dyskeratosis congenita in the NHLBI Exome Sequencing Project [52]. Despite these limitations, our findings that A279T modulates canonical as well as non-canonical telomerase activities highlight the complexity of telomerase expression in normal cellular homeostasis and human diseases. Whereas the mechanisms underlying our observations have not been fully delineated, our current findings support additional larger, case control studies to define the frequency and clinical significance of A279T expression in esophageal carcinomas and related preneoplastic lesions.

## Supporting Information

Figure S1 **Sanger's sequencing (left panel) and pyrosequencing (right panel) of homozygous, heterozygous, and wild type A279T.**
(TIF)

Figure S2 **Immunoblot demonstrating no appreciable decrease in β-catenin levels in HeLa cells expressing TERT variant A1062T.**
(TIF)

Table S1 **Primers and PCR Conditions.**
(DOCX)

Table S2 **Real-Time Quantitative RT-PCR Primers and Antibodies.**
(DOCX)

Table S3 **Real-Time PCR Analysis of TERT and TERC Expression in Cell Lines.** (relative copy/# β-actin x e4).
(DOCX)
