# Supplementary figures and images for: Telomerase Variant A279T Induces Telomere Dysfunction and Inhibits Non-Canonical Telomerase Activity in Esophageal Carcinomas
Source: PLoS One. 2014 Jul 1;9(7):e101010. doi: 10.1371/journal.pone.0101010 (PMC4077737; doi:10.1371/journal.pone.0101010)

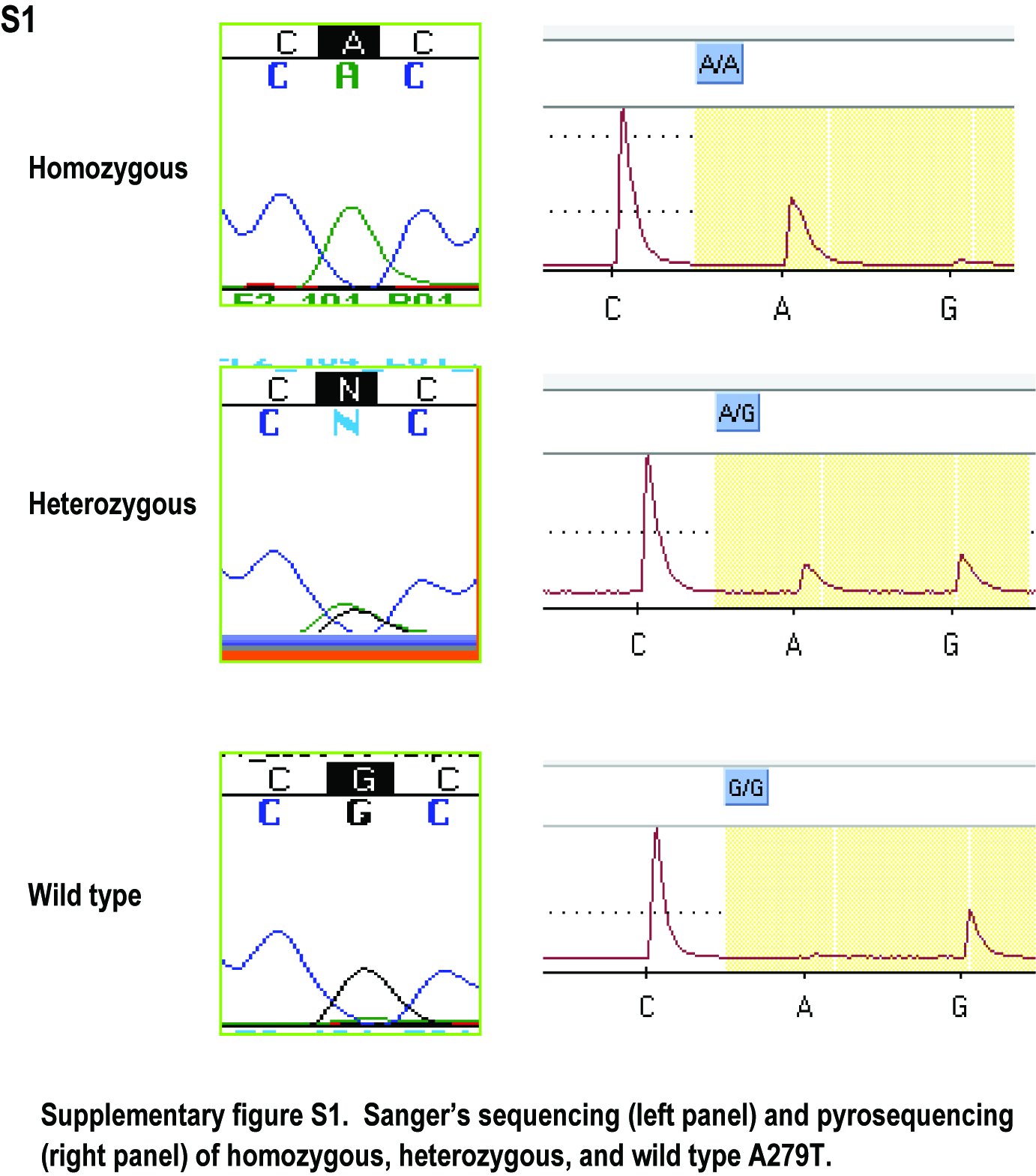

Supplement: Figure S1 — Sanger's sequencing (left panel) and pyrosequencing (right panel) of homozygous, heterozygous, and wild type A279T. (TIF) [file pone.0101010.s001.tif]

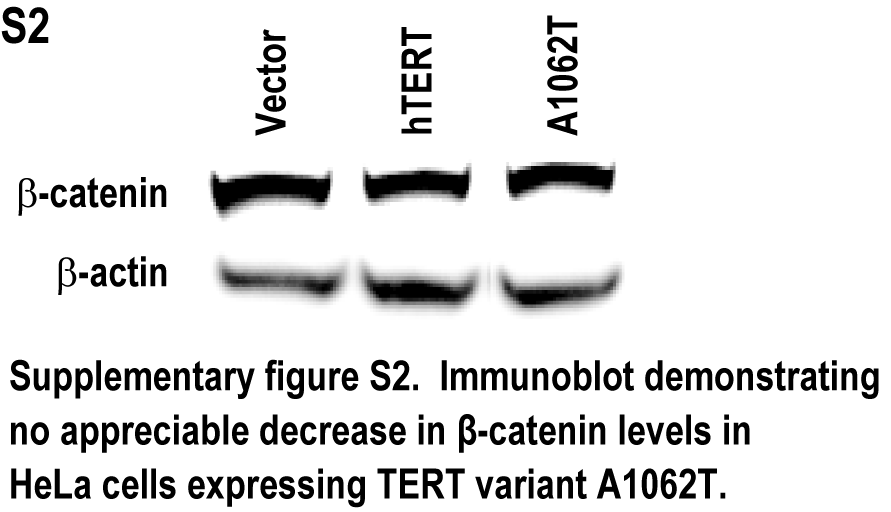

Supplement: Figure S2 — Immunoblot demonstrating no appreciable decrease in β-catenin levels in HeLa cells expressing TERT variant A1062T. (TIF) [file pone.0101010.s002.tif]
