# Supplementary material for: Telomerase Variant A279T Induces Telomere Dysfunction and Inhibits Non-Canonical Telomerase Activity in Esophageal Carcinomas
Source: PLoS One. 2014 Jul 1;9(7):e101010. doi: 10.1371/journal.pone.0101010 (PMC4077737; doi:10.1371/journal.pone.0101010)
Supplement: Table S1 — Primers and PCR Conditions. (DOCX) [file pone.0101010.s003.docx]

**Supplementary Table S1**: **Primers and PCR Conditions**

| **Assay** | **Gene** | | **Exon** | | **Mutation** | | **Primer sequence / catalogue number** | **Annealing Temperature (˚C)** | |  |
| --- | --- | --- | --- | --- | --- | --- | --- | --- | --- | --- |
|  |  | |  | |  | |  |  | |  |
| Pyrosequencing | wt TERT | | 2 | | A279T | | PCR primers: | 65-60 | |  |
|  |  | |  | |  | | F: Biotin- GGTGGCCGCGATGTGGAT |  | |  |
|  |  | |  | |  | | R: AACCGTGGTTTCTGTGTGGTGTC |  | |  |
|  |  | |  | |  | | Sequencing primer: |  | |  |
|  |  | |  | |  | | GTGTCACCTGCCAGA |  | |  |
|  |  | | 15 | | A1062T | | PCR primers: | 65-63 | |  |
|  |  | |  | |  | | F: CTGTTTTCCCCCAGGGATGT |  | |  |
|  |  | |  | |  | | R: Biotin- CGAGTCAGCTTGAGCAGGAATG |  | |  |
|  |  | |  | |  | | Sequencing primer: |  | |  |
|  |  | |  | |  | | CGAGTCAGCTTGAGCAGGAATG |  | |  |
| Lentiviral cloning | | TERT | |  | |  | F: CACCATGCCGCGCGCTCCCCGCTGC | |  | |
|  | |  | |  | |  | R: TCCGTCCAGGATGGTCTTGAAGTC | |  | |
|  | | TERC | |  | |  | F: CACCGGGTTGCGGAGGGTGGGCCTG | |  | |
|  | |  | |  | |  | R: GCATGTGTGAGCCGAGTCCTG | |  | |
| FISH | telomere repeat | | | | | | CCCTAACCCTAACCCTAA |  | |  |
|  | CENP-B binding sequence | | | | | | ATTCGTTGGAAACGGGA |  | |  |
|  |  | |  | |  | |  |  | |  |
