# Supplementary material for: Telomerase Variant A279T Induces Telomere Dysfunction and Inhibits Non-Canonical Telomerase Activity in Esophageal Carcinomas
Source: PLoS One. 2014 Jul 1;9(7):e101010. doi: 10.1371/journal.pone.0101010 (PMC4077737; doi:10.1371/journal.pone.0101010)
Supplement: Table S2 — Real-Time Quantitative RT-PCR Primers and Antibodies. (DOCX) [file pone.0101010.s004.docx]

**Supplementary Table S2: Real-Time Quantitative RT-PCR Primers and Antibodies**

| **Assay** | **Gene/protein** | **Company** | **Catalogue #** |
| --- | --- | --- | --- |
| qRT-PCR | CCND1 | Applied | Hs99999004_m1 |
|  | Dkk-1 | Biosystems | Hs00183740_m1 |
|  | TERT |  | Hs00972656_m1 |
|  | TERC |  | Hs03454202_s1 |
|  | CCND1 |  | Hs00765553_m1 |
|  | CASP8 |  | Hs01018151_m1 |
|  | p57 |  | Hs00175938_m1 |
|  | IL-6 |  | Hs00985639_m1 |
|  | IL-8 |  | Hs00174103_m1 |
|  | BRCA1 |  | Hs01556193_m1 |
|  | BRCA2 |  | Hs00609073_m1 |
|  | JUNB |  | Hs00357891_s1 |
|  | TNF |  | Hs01113624_g1 |
|  | β-actin - forward | GCGAGAAGATGACCCAGATC | |
|  | β-actin - reverse | CCAGTGGTACGGCCAGAGG | |
|  | β-actin - probe | 6FAM-CCAGCCATGTACGTTGCTATCCAGGC-TAMRA | |
|  |  |  |  |
| Western blot/IP/ | β-catenin | Abcam | ab16051 |
| IHC | vinculin | Millipore | 90227 |
|  | β-tubulin | Abcam | ab6046 |
|  | F-actin | Abcam | ab205 |
|  | CDH1 | Cell signaling | 4065 |
|  | BRG1 | Millipore | 07-478 |
|  | TERT | Rocklands | 600-4C1-252S |
|  | β-galactosidase | Abcam | ab9361 |
|  | GAPDH | Abcam | ab9484 |
|  | TIN2 | Abcam | ab13791 |
|  | TPP1 | Abcam | ab54685 |
|  | RAP1 | Abcam | ab14404 |
|  | TRF1 | Abcam | ab105709 |
|  | TRF2 | Abcam | ab4182 |
|  | Pot1 | Abcam | ab21382 |
|  | Ki67 | Dako | M7240 |
|  |  |  |  |
|  |  |  |  |
