# Supplementary material for: Telomerase Variant A279T Induces Telomere Dysfunction and Inhibits Non-Canonical Telomerase Activity in Esophageal Carcinomas
Source: PLoS One. 2014 Jul 1;9(7):e101010. doi: 10.1371/journal.pone.0101010 (PMC4077737; doi:10.1371/journal.pone.0101010)
Supplement: Table S3 — Real-Time PCR Analysis of TERT and TERC Expression in Cell Lines. (relative copy/# β-actin x e4). (DOCX) [file pone.0101010.s005.docx]

**Supplementary Table S3: Real-Time PCR Analysis of TERT and TERC Expression in Cell Lines (relative copy # / β-actin x e4)**

|  | **TERC** | | **TERT** | |
| --- | --- | --- | --- | --- |
|  | **Ct** | **Copy #** | **Ct** | **Copy #** |
| **A549** | 22.2 | 11037 | 28.4 | 11 |
| **Calu-6** | 24.5 | 1337 | 31.3 | 1 |
| **H841** | 24.4 | 1035 | 26 | 39 |
| **H1299** | 21.5 | 14181 | 27.9 | 12 |
| **H2126** | 22 | 10309 | 30 | 3 |
| **H322** | 22.3 | 6859 | 29 | 5 |
| **H358** | 21.5 | 15234 | 29.8 | 3 |
| **H2087** | 22.3 | 10549 | 31.3 | 1 |
| **EsC1** | 22.4 | 5258 | 29.3 | 3 |
| **EsC2** | 23.7 | 2191 | 29.3 | 4 |
| **293FT** | 22 | 32532 | 27.7 | 47 |
| **HBEC *** | 24 | 41012 | 21.3 | 36732 |
| **NHBE** | 24.7 | 2875 | 35.5 | 0 |
| **BEAS** | 22.8 | 10477 | 27.1 | 47 |
| **Saos2** | 24.5 | 2299 | 36.6 | 0 |
| **Het1A** | 22.7 | 13659 | 29 | 0 |
| **Met5a** | 24.9 | 4948 | 35.2 | 14 |
| **VA13** | 36 | 0 | 36.3 | 0 |
| **SAEC** | 23.2 | 4523 | 36.2 | 0 |

* Immortalized with Cdk4 and hTERT.
